# Multifunctional NADES-Based Extracts from *Paeonia lactiflora Pall.* Flowers for Potential Cosmetic and Pharmaceutical Applications

**DOI:** 10.3390/molecules31010097

**Published:** 2025-12-25

**Authors:** Carla Villa, Eleonora Russo, Anna Maria Schito, Francesco Saverio Robustelli della Cuna, Cristina Sottani, Marta Barabino, Debora Caviglia

**Affiliations:** 1 Department of Pharmacy (DIFAR), Section of Drug and Cosmetic Chemistry, University of Genoa, 16132 Genoa, Italy; eleonora.russo@unige.it (E.R.); martabarabino.ge@gmail.com (M.B.); debora.caviglia@edu.unige.it (D.C.); 2 Department of Surgical Sciences and Integrated Diagnostics (DISC), University of Genoa, 16132 Genoa, Italy; anna.maria.schito@unige.it; 3 Environmental Research Center, Istituti Clinici Scientifici Maugeri IRCCS, 27100 Pavia, Italy; saverio.robustelli@icsmaugeri.it (F.S.R.d.C.); cristina.sottani@icsmaugeri.it (C.S.)

**Keywords:** NADES extraction, microwaves, green cosmetic multifunctional ingredient, *Paeonia lactiflora* extract

## Abstract

*Paeonia lactiflora Pall.* is a perennial herbaceous plant widely renowned for its floral ornamental appeal, distinctive pleasant scent, and utilization in folk remedies. Roots and barks are traditionally used in Chinese medicine for various properties, including anti-inflammatory, antioxidant, antibacterial, anticancer, cardiovascular, and neuroprotective effects. Considering the growing interest and demand in the pharmaceutical and cosmetic fields for sustainable and bioactive botanical derivatives, this study aimed to apply NADES (natural deep eutectic solvents) extraction on fresh flowers of *Paeonia lactiflora Pall.* The purpose was to obtain a natural, multifunctional, and ready-to-use cosmetic ingredient with concurrent antioxidant activity, antimicrobial functionalities, and olfactive properties. The eutectic systems selected in this study were composed of betaine as the hydrogen bond acceptor and glycerol and/or sorbitol as the hydrogen bond donors. These eutectic systems under microwave activation led to a rapid extraction, from peony fresh flowers, of considerable phenolic amounts (from 33.0 to 34.4 mg of gallic acid equivalents per gram of fresh flowers), which confer to the whole NADES-based extract an excellent radical scavenging activity (around 87.5%, compared to Trolox) and a pleasant fragrance, due to the extraction of some characteristic volatile compounds, as confirmed by GC-MS analysis. Antimicrobial assays against different Gram-positive and Gram-negative strains demonstrated good inhibitory activity of the sample against multidrug-resistant *Staphylococcus* species (MIC ranging from 0.9 to 14.5 mg/mL) and against *Enterococcus* species (from 28.8 to 57.8 mg/mL). Furthermore, results on different *Staphylococcus aureus* strains disclosed additional interesting anti-biofilm properties. Preliminary long-term studies (up to 9 months) on these combined properties highlighted the stabilizing effect of NADESs on the active metabolites, confirming their potential as natural and functional ingredients that could be directly incorporated into pharmaceutical and cosmetic formulations, offering enhanced efficacy and improved stability.

## 1. Introduction

The genus *Paeonia*, the sole member of the *Paeoniaceae* family, is widely renowned for its ornamental appeal, scented flowers, and long history of utilization in folk remedies [1]. China is the region of its origin and evolution [2], but at present, this genus is also widespread in the other temperate regions of Asia, Europe, and North America [3,4]. Many species have been studied for their medicinal properties [5], in particular, *Paeonia lactiflora Pall. (P. lactiflora*), whose root is traditionally used in Chinese medicine and appears in Chinese pharmacopeia monographs [6].

A review of the extant literature indicates that *P. lactiflora* demonstrates considerable potential in numerous domains of research [7]. Rhizomes, barks, and aerial parts have been investigated, disclosing the presence of a large number of compounds (terpenoids, polyphenols, flavonoids, organic acids, and other compounds) with different biological activities, including anti-inflammatory, antioxidant, antibacterial, anticancer, cardiovascular, and neuroprotective properties [8,9,10]. Flowers in particular, often discarded as waste in the cultivation of medicinal peony [11], present antioxidant and antibacterial properties [12], being rich in polyphenols, phenolic acids, anthocyanins, monoterpene glycosides, and flavonoids, useful in topical formulations.

As regards dermocosmetic uses, *P. lactiflora* flower extracts have been added to skin care products for anti-aging and bleaching effects [13,14,15]. Moreover, according to the European Commission’s CosIng (Cosmetic Ingredient Database) [16], extracts of *Paeonia lactiflora* from stems, leaves, flowers, and callus culture biomass are recognized as natural and interesting cosmetic ingredients. In fact, in a context of sustainability, the focus of dermocosmetic research has consolidated around the development of innovative products based on natural bioactive ingredients, derived from renewable sources, with a preference for those obtained through environmentally friendly extraction methods.

In consideration of the aforementioned statements and in the context of our green cosmetic and pharmaceutical objectives [17,18], the aim of this study was to explore the dermocosmetic potential of peony flower extracts when exploiting the advantages and benefits of natural deep eutectic solvents (NADESs), efficient alternative green solvents for the extraction of several bioactive metabolites from plants [19,20]. Often considered the green solvents for the 21st century [21], NADESs are primarily formed from a mixture of major plant metabolites (i.e., organic acids, sugars, alcohols and polyols, amino acids, and quaternary ammonium salts), combining hydrogen bond donors (HBDs) and hydrogen bond acceptors (HBAs) in a eutectic system (often liquid at room temperature), which produces a highly stable hydrogen-bonding network. They are characterized by favorable properties and advantages over conventional organic solvents (low vapor pressure, tunable polarity for enhanced extractive capacity, low production costs, etc.). In addition, they possess the remarkable ability to act as storage media for the extracted phytochemicals, thus preventing their degradation. Moreover, due to their polarity, they are suitable solvents to be used in combination with microwave (MW) activation for improving sustainable extraction performance [22,23].

The rationale for the utilization of such eutectic systems in our study can also be attributed to the fact that many NADES components are still employed as cosmetic ingredients and pharmaceutical excipients for their intrinsic properties [19]. Therefore, once their stability [24] and safety (i.e., contaminations due to possible coextractions) [25] are assessed, the NADES-based extracts could be incorporated directly into the final formulation as ready-to-use ingredients [26], thus obviating the requirement for costly separation or purification steps and enhancing the overall performance.

In accordance with a previously published procedure [27], in this study, *P. lactiflora* fresh flowers were extracted by microwave activation as an alternative energetic source [22] using two different NADES systems (containing betaine, glycerin, and sorbitol).

The NADES-based samples were characterized in terms of total phenolic content (TPC) recovery [28], radical scavenging activity (RSA) [29], and antimicrobial properties [30], with a particular focus on the evaluation of the minimum inhibitory concentration (MIC) [31] and anti-biofilm properties (time-kill and antibiofilm tests) [32,33,34]. Moreover, its volatile fraction, characterized by a distinctive aromatic profile, was analyzed by gas chromatography–mass spectrometry (GC-MS) [35]. As in previous studies [27,36], preliminary long-term stability studies were carried out to investigate the real potential of the NADES-based extract as an active cosmetic ingredient. With this purpose, the values obtained for TPC, RSA%, and MIC were reassessed, following a nine-month storage period at room temperature.

## 2. Results

### 2.1. MW NADES Extraction

Following a previously published MW procedure [27], two extracts (PF-BG and PF-BGS) were obtained from the corresponding NADESs (BG-betaine/glycerol and BGS-betaine/glycerol/sorbitol) within five minutes, after a simple filtration, from fresh flowers of *P. lactiflora*. Considering solvents and peony metabolites extracted as a complex of concurrent active compounds, the NADES-based samples were directly analyzed and used without the need to isolate the extracted molecules.

The polarity of the NADES components, when combined with the dielectric heating of microwave irradiation, resulted in the swift attainment and sustenance of the target process temperature setpoint (75 °C). This occurrence transpired within a time frame of merely 10–15 s. The constant temperature was then maintained through power modulation within the range of 0–300 watts. An inherent continuous feedback mechanism within the MW prototype meticulously controlled this process, thereby preventing overheating and the subsequent thermal degradation of the botanical matrix and extracts.

### 2.2. Total Phenolic Content (TPC) and Radical Scavenging Activity (RSA%)

Table 1 summarizes the results related to the total phenolic content (TPC) and percentage of radical scavenging activity (RSA%) for the NADES-based extracts obtained. Both samples (PF-BG, PF-BGS) demonstrated strong antioxidant activity (around 87%, with respect to Trolox as the positive control), consistent with the corresponding TPC values, expressed as milligrams of gallic acid equivalents per gram (mg GAE/g) of fresh leaves (34.38 and 33.78 mg GAE/g for PF-BG and PF-BGS, respectively).

### 2.3. Minimal Inhibitory Concentrations (MICs)

The antibacterial activity of the NADES-based extracts (PF-BG and PF-BGS) and the corresponding eutectic solvents (BG and BGS) was evaluated by calculating the Minimal Inhibitory Concentrations (MICs) against 38 bacterial isolates, according to the guidelines of the European Committee on Antimicrobial Susceptibility Testing (EUCAST) [31]. They are representative of clinically relevant multidrug-resistant Gram-positive (32 strains) and Gram-negative (6 strains) species (see Section 4.4). Both samples disclosed variable levels of activity against all the Gram-positive species considered, while they resulted in inactivity against all Gram-negative strains tested.

Table 2 presents the MIC values of both NADES-based samples, PF-BG and PF-BGS, assessed against all the susceptible species considered. PF-BG seems to have a general, greater antibacterial potency and a broader spectrum of activity when compared to PF-BGS. As regards *S. aureus*, MIC values of PF-BG fall within a range from 7.2 to 14.4 mg/mL (PF-BGS: from 14.4 to 57.6 mg/mL), and in the case of *S. epidermidis,* from 3.6 to 7.2 mg/mL (PF-BGS: from 14.4 to 28.8 mg/mL). Even in the comparative study of the coagulase-negative species, sample PF-BG performs better than PF-BGS with MIC values from 0.9 to 14.4 mg/mL (PF–BGS from 7.2 to 57.6 mg/mL). The most sensitive species to this NADES-based extract were found to be *S. saprophyticus*, *S. warneri*, *S. hominis,* and *S. auricularis* (MIC 0.9 mg/mL), followed by *S. capitis* (MIC 1.8 mg/mL), *S. simulans* (MIC 7.2 mg/mL), *S. lugdunensis,* and *S. haemolyticus* (MIC 14.4 mg/mL). Moreover, PF-BG shows a lower but meaningful performance against the *Enterococcus* genus (28.8 mg/mL against *E. faecalis*, 57.6 mg/mL against *E. faecium* isolates), while the PF-BGS sample seems to be inactive. Neither NADESs, BG, nor BGS displayed any activity across the entire range of concentrations studied.

In view of the results obtained, disclosing the optimum level of activity of PF-BG, this sample was selected for further investigations to ascertain the extent of its antimicrobial efficiency.

### 2.4. Time-Kill Test

**Figure 1:**
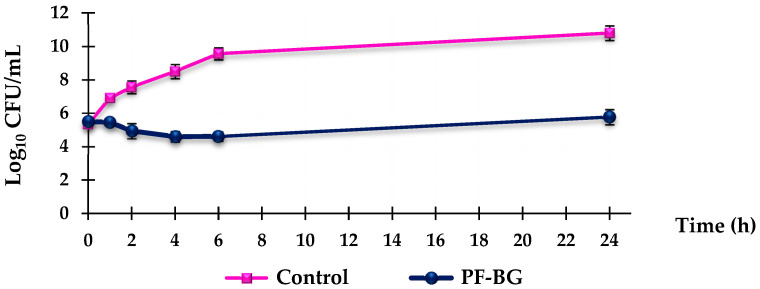
Time-kill curve for PF-BG performed at a concentration of 4× MIC on *S. aureus* 18 (MRSA) across a 24 h test against bacterial growth control.

To determine the antibacterial mechanism of action of the extract, time-kill assays were performed on *S. aureus*, as the most clinically relevant species. For this purpose, bactericidal curves were obtained, studying three selected strains (18, B, and ATCC 29213) at a concentration four times higher than the MIC for 24 h. All strains, despite displaying different resistance phenotypes, showed overlapping results, exemplified in Figure 1 by the 18 MRSA strains. As shown, strain treatment with PF-BG revealed the extract’s bacteriostatic activity at 24 h, essentially maintaining the bacterial load of the initial inoculum after a slight reduction in the initial bacterial concentration during the first 6 h.

### 2.5. Biofilm Inhibition

The potential of the PF-BG sample in preventing the biofilm formation by *S. aureus* was subsequently assessed, according to Cramton et al. [32], on seven selected clinical and reference strains, namely B, 10, 11, 18, 189, N2, and ATCC 29213.

Assuming a potential cosmetic use of the PF-BG extract as a preservative against Gram-positive strains, its inhibitory potency was compared with that of Sodium Dehydroacetate (SDA), a commercial broad-spectrum antimicrobial agent against bacteria, yeasts, and molds, authorized by the European Cosmetics Regulation 1223/09 as a preservative ingredient (Annex 5, reference n. 13), at a maximum concentration of 0.6% [37].

For the determination of SDA biofilm inhibition, the MIC values of SDA against the 7 selected *S. aureus* strains (required for carrying on the experiments) were previously assessed (Table 3), using different dilutions of the sample in the same solvent (BG) (also tested).

**Figure 2:**
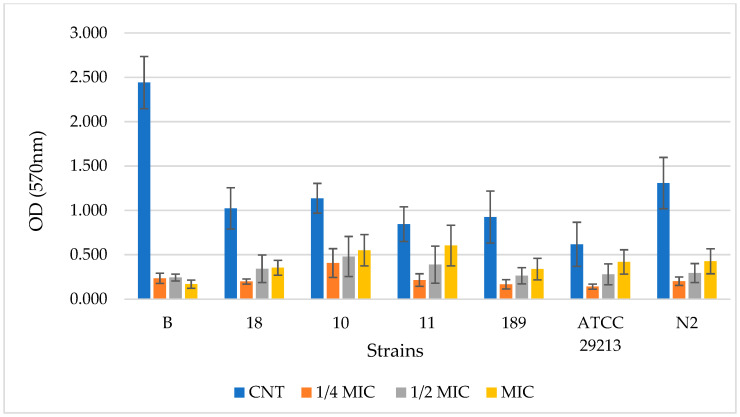
Inhibitory effect of PF-BG in the biofilm formation assay on different strains of *S. aureus* obtained by the crystal violet (CV) method. Biofilm biomass was quantified by measuring the optical density (OD) of the CV after its solubilization in ethanol at 570 nm. Treatments include an untreated control (CNT, blue), MIC (yellow), ¼ MIC (orange), and ½ MIC (gray). Data are reported as the mean value of four independent experiments (*n* = 4).

**Figure 3:**
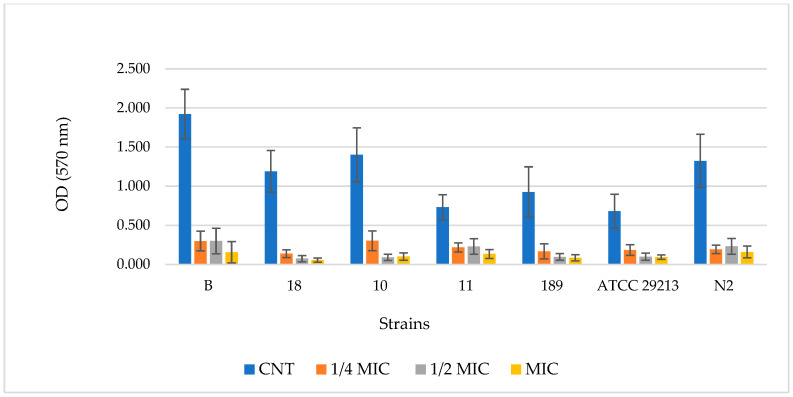
Inhibitory effect of SDA in the biofilm formation assay on different strains of *S. aureus* obtained by the crystal violet (CV) method. Biofilm biomass was quantified by measuring the optical density (OD) of the CV after its solubilization in ethanol at 570 nm. Treatments include an untreated control (CNT, blue), MIC (yellow), ¼ MIC (orange), and ½ MIC (gray). Data are reported as the mean value of four independent experiments (*n* = 4).

Figure 2 and Figure 3 display the results related to the quantified biofilm biomass formation obtained by the crystal violet assay [33] for BF-BG and SDA, respectively. Data are expressed as the optical density (OD) of each well stained with crystal violet, measured at 570 nm using a microtiter-plate reader. The OD of each selected strain containing 3 different PF-BG and SDA concentrations (¼ MIC. ½ MIC, and MIC), respectively, in comparison to the control (strain without sample), is reported.

In regard to PF-BG, for all strains, the CNT sample is significantly different from ¼ MIC, ½ MIC, and MIC (*p* < 0.0001 ****). Only for strain 11 is there also a significant difference for ¼ MIC vs. MIC and for ½ MIC vs. MIC. For strain AT20213, there is also a significant difference for ¼ MIC vs. MIC (*p* < 0.001 ***).

Concerning SDA, for all strains, the CNT sample is significantly different from ¼ MIC, ½ MIC, and MIC (*p* < 0.001 ***); for all strains, ¼ MIC, ½ MIC, and MIC are not significantly different.

For a better comprehension of the results obtained in Table 4 and Table 5, data were converted into the corresponding inhibition percentage values (calculated by Equations (2) and (3)) for each strain at the three concentrations considered in the study (¼ MIC, ½ MIC, and MIC).

The PF-BG sample demonstrated the capacity to inhibit biofilm formation across all analyzed *S. aureus* strains, notably even at concentrations below the MIC. In particular, strain B, an excellent biofilm producer, showed a remarkable reduction in biofilm formation in the presence of PF-BG, an effect that was very similar across the three tested concentrations, with an inhibition percentage ranging between 90 and 93%. This behavior differs from that observed for the remaining six strains analyzed, for which a decrease in inhibitory capacity is observed as the concentration of PF-BG increases. For these strains, inhibition at ¼ MIC ranged between 64% (strain 10) and 85% (strain N2), at ½ MIC between 54% (strain 11) and 76% (strain N2), and at MIC between 29% (strain 11) and 67% (strain N2). Therefore, along with the heterogeneous ability of the different strains to produce biofilm, susceptibility to the PF-BG extract was also found to be strain-dependent, with isolate B being very susceptible, strains 18, 189, and N2 showing intermediate susceptibility, and the remaining isolates, 10, 11, and ATCC 29213, showing lower sensitivity. Identical concentrations of the solvent BG, when tested on the same strains of *S. aureus*, proved completely ineffective in inhibiting biofilm formation.

As reported in Table 5, the SDA compound, characterized by MIC values comparable to those of PF-BG, demonstrated a slightly better antibiofilm potency than the NADES-based extract, and, also in this case, was heterogeneous among the different strains tested. In particular, inhibition at ¼ MIC ranged between 70 and 89%, between 69 and 94% at ½ MIC, and between 82 and 96% at MIC. Although at MIC values, SDA has shown greater effectiveness in reducing biofilm formation by the strains considered compared to PF-BG, it is certainly noteworthy that at sub-MIC values, particularly at ¼ MIC, the antibiofilm potency of PF-BG and SDA is essentially comparable.

### 2.6. GC-MS Analysis

**Figure 4:**
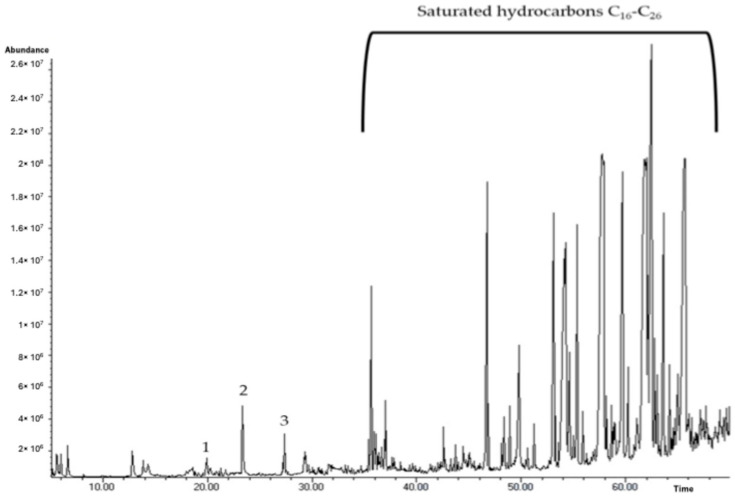
Typical chromatogram of the volatile fraction of the NADES-based sample PF-BG. Attribution of the aromatic compounds of interest: 1 = limonene, 2 = 2–phenylethanol, 3 = citronellol.

The GC-MS of the volatile fraction of PF-BG, the most promising sample, was performed as a preliminary analysis in order to verify and attribute the scented character of the NADES-based extract. The typical chromatogram is reported in Figure 4. The analysis led to the identification of 34 components, with an identification rate of 38%. As shown in Figure 4, the compounds most represented in the extract were the saturated hydrocarbons from C_16_ to C_26_, as already documented in a previous analytical study concerning the volatile fraction of *P. lactiflora* [35]. Only three typical and characteristic terpenoids, commonly used as fragrances, were isolated and identified: a monocyclic monoterpene (d-limonene, 0.02%) and two oxygenated derivatives (2-phenylethanol and citronellol, 0.62% and 0.15%, respectively). Analytical results and olfactory characters are reported in Table 6.

### 2.7. Preliminary Long-Term Stability Studies

In order to verify the ability of the eutectic solvent to maintain the properties of the sample over time [42] and with a view to obtaining basic information about the actual applicability of the sample as an ingredient for future cosmetic applications, a preliminary long-term stability study was conducted on the PF-BG sample in relation to TPC, RSA, and MIC values. Consequently, instrumental analyses were repeated in regard to the Folin–Ciocalteu and the DPPH assay, in accordance with the procedures previously outlined, after 90 days (t90) and up to 9 months (t180). The antibacterial activity was then re-evaluated after a period of 90 days by determining the MIC on selected bacterial strains of *S. aureus*, *S. epidermidis*, *E. faecalis*, and *E. faecium*, according to the protocol described above. The results of the study are set out in Table 7 and Table 8.

TPC and RSA values for PF-BG are not significantly different between t_0_ and t_90_, while significant differences are recorded between t_0_ and t_180_ and between t_90_ and t_180_.

The investigation of the MIC after 90 days showed a coherent behavior, maintaining the antimicrobial activity constant against the strains analyzed.

## 3. Discussion

The aim of the research was to enhance the value of peony flowers by means of a simple, environmentally friendly extraction process, thereby obtaining a multifunctional ingredient that was ready for use [26]. It is important to note that the extraction solvents are readily available and present a negligible risk. Moreover, they add a cosmetic value to the complex, having their own functionality, being commonly used as humectants and moisturizing agents.

The procedure is in accordance with the principles of green extraction [43], taking into account the utilization of environmentally friendly solvents, an alternative energy source (MW), and the selection of a plant matrix that could be regarded not only as a renewable source but, in some cases, as actual processing waste (in the cultivation of the root of the medicinal peony).

As stated in the previous literature [29], the suitable eutectic mixtures were evaluated to enhance the contemporaneous extraction of lipophilic and hydrophilic compounds with diverse functionalities (i.e., antioxidant, hydrophilic polyphenols, and hydrophobic volatile compounds with a pleasant olfactory character).

A significant benefit of this kind of extraction is that the resulting sample can be analyzed directly, obviating the necessity for additional purification. Indeed, the sample is prepared for analysis and subsequent utilization following straightforward filtration. The product is then treated as if it were the ultimate ingredient ready to be incorporated into a formulation.

In order to ensure the accuracy of evaluations of phenolic amount in NADES-based extracts (particularly with FC assay), it is imperative to consider the potential interference of components present in NADESs, such as sorbitol (a sugar derivative), which can lead to an overestimation of the phenolic content that cannot be predicted. Consequently, NADES-based blanks were meticulously prepared and utilized in the analysis to account for any potential alterations in absorption caused by the NADES itself.

Notwithstanding the results obtained regarding the proven antioxidant activity, an interesting and remarkable finding concerns the microbiological properties of the extract.

The microbiological evaluation of the NADES-based extracts PF-BG and PF-BGS revealed their promising antibacterial properties against various Gram-positive multi-resistant strains of species belonging to the genera *Staphylococcus*, including MRSA and MRSE isolates, and *Enterococcus* ones.

Although the total phenolic content (TPC) and the radical scavenging activity (RSA%) of both NADES-based extracts were comparable, the PF-BG sample showed superior efficacy compared to PF-BGS in terms of minimum inhibitory concentrations (MIC values for PF-BG were consistently lower than those of PF-BGS) and a broader spectrum of action, being active even against two enterococcal species, such as *E. faecalis* and *E. faecium*. Noteworthy is the fact that both extracts, although with different potencies, showed excellent activity also against a wide number of coagulase-negative *Staphylococcus* species, with MICs up to 0.91 mg/mL for *S. saprophyticus*, *S. warneri*, *S. hominis*, and *S. auricularis.*

Despite the MIC values appearing elevated in comparison to those of recognized antimicrobials employed in cosmetic products (such as parabens) [44], these data are of relevant pertinence. It is imperative to acknowledge that the concentrations (mg/mL) pertain to the extracted NADES complex, wherein the solvent constitutes the preponderance of the sample and was found to be ineffectual at all concentrations for all the strains under investigation. Neither PF-BG nor PF-BGS showed activity against Gram-negative strains included in this study. This limitation is consistent with the intrinsic resistance mechanisms of Gram-negative bacteria, mainly due to the presence of the outer membrane, an extremely impermeable structure that can hinder the penetration or retention of NADES-based hydrophilic compounds. For the above-mentioned reasons, and due to the lower viscosity of the PF-BG sample compared to PF-BGS, which makes it easier to handle, further microbiological studies were conducted only on the former extract. Time-kill kinetics, subsequently performed on selected MRSA strains, confirmed the bacteriostatic nature of PF-BG, capable of maintaining the initial bacterial inoculum stable for 24 h. This suggests that PF-BG at 4× MIC may inhibit bacterial proliferation without inducing cell lysis, a property that could be advantageous in reducing inflammatory responses in potential topical applications. The NADES-based extract also inhibited biofilm formation in all selected *S. aureus* strains. This inhibition was particularly consistent (90–93%) at the various concentrations tested for strain B, an excellent biofilm producer whose MIC was higher than those of the remaining strains, for which the biofilm inhibition tendency was different and variable. In fact, for the remaining six strains, inhibition ranged from 64% to 85% at ¼ MIC and decreased at higher MIC (inhibition between 29% and 67%). This behavior could suggest a possible hormetic effect of the sample, a biphasic dose–response in which low amounts induce a stronger biological response compared to higher doses, which may instead reduce efficacy by triggering stress adaptation. This inverse trend in the dose response, observed in different strains, might reflect complex mechanisms of interaction between the components of PF-BG and the regulatory pathways of biofilm, potentially involving disruption of quorum sensing or interference with the synthesis of extracellular polymeric substance, which is crucial for biofilm formation. The strain-dependent variability in response further highlights the need for mechanistic studies to clarify the molecular targets of PF-BG. The comparative analysis with Sodium Dehydroacetate (SDA) revealed that the NADES-based extract possesses antibiofilm activity comparable to that of the preservative at sub-MIC levels (**¼** MIC). This outcome is of pertinence when considering the comparison of a pure molecule with a natural complex consisting of a solvent (NADES) and extracted active metabolites. Therefore, although SDA was slightly more effective at MIC, the similar performance of PF-BG at lower dosages highlights its potential as a natural alternative for cosmetic applications, especially when biofilm control is critical.

Regarding GC-MS analysis, as might be expected, the number of scented volatile compounds was very low. Only limonene, 2-phenylethanol, and citronellol were detected in the NADES extract. Nonetheless, their presence gives an interesting olfactory contribution to the sample for a pleasant and distinctive fragrance.

Preliminary long-term stability studies have highlighted the ability of NADESs to act as metabolite stabilizers; this has allowed the PF-BG sample to maintain its antioxidant activity and antibacterial capacity even after 90 days. These preliminary results indicate the promising applicability of the compound, which therefore warrants further in-depth and dedicated studies for a commercial purpose.

## 4. Materials and Methods

### 4.1. Plant Material

Fresh peony flowers (*Paeonia Lactiflora Pall*) were collected in the Ligurian hinterland (Tiglieto, Liguria, Italy, 44°30′05.5″ N 8°35′47.5″ E) in May 2024 by a local supplier. Samples were immediately refrigerated at +4 °C and subsequently stored at −20 °C until extraction. Plants were identified according to Pignatti’s method [45]. Voucher specimens (LP01, LP02) were kept in the Department of Pharmacy of the University of Genova, Italy.

### 4.2. Chemicals

Betaine (purity grade ≥ 99%), sorbitol (purity grade ≥ 99%), glycerine (purity grade ≥ 98%), DPPH (2,2-diphenyl-1-picrylhydrazyl), Trolox (6-hydroxy-2,5,7,8-tetramethylchroman-2-carboxylic acid) (purity grade ≥ 98%), Folin–Ciocalteu phenol reagent, Sodium Dehydroacetate (SDA—purity grade ≥ 99%), and gallic acid (purity grade ≥ 98%) were purchased from Sigma-Aldrich (Milan, Italy).

A Milli-Q system (Millipore SA, Molsheim, France) was used to produce freshly prepared, redistilled water.

NADESs were prepared on-site in the laboratory according to an MW method [3] suitably modified. NADES abbreviations and compositions are reported as follows:

BG = Betaine/Glycerol (1:2 molar ratio).

BGS = Betaine/Glycerol/Sorbitol (3:2:2 molar ratio).

### 4.3. Apparatus

MW multimode prototype (emitted power max: 900 watts), equipped with a specially designed Pyrex reactor, a magnetron operating at 2.45 GHz, two optical fiber probes for temperature measurement, and a control unit that allows managing different process parameters such as emitted power, temperature setpoint, and mechanical stirring.

Ultrasonic Cleaner Transonic 130 C.R. (ACAD Pharmaceutical Inc., Basel, Switzerland).

Thermoscientific UV/VIS spectrophotometer (Evolution 300, Fischer Scientific, GmbH, Schwerte, Germany).

GC/MS analyses were carried out using a GC Model 6890N, coupled to a benchtop MS Agilent 5973 Network (Agilent, Santa Clara, CA, USA), using an Elite-5MS (5% phenyl methyl polysiloxane) capillary column of 30 m × 0.32 mm i.d. and a 0.32 μm thick film (Agilent, Santa Clara, CA, USA).

IMark Microplate reader (Bio-Rad Laboratories Inc., Hercules, CA, USA).

### 4.4. Bacterial Strains

In this study, 38 multidrug-resistant (MDR) bacterial clinical strains, consisting of 32 Gram-positive isolates and 6 Gram-negative strains, were used. Among Gram-positive organisms, 11 strains belong to the *S. aureus* species, including 9 methicillin-resistant (MRSA) (no. 10, 11, 18, 187, 189, A, B, C, and D), 1 reference strain (*Staphylococcus aureus subsp aureus*-ATCC® 29213™), and 1 methicillin-susceptible (N2) strain. Six strains of *S. epidermidis* were also included (n° 22, 180, 181, 222, 198, 216), all methicillin-resistant (MRSE), five of which were also resistant to linezolid. Nine strains belong to other *Staphylococcus* species, including *S. saprophyticus* (n°. 41), *S. capitis* (n°. 71), *S. warneri* (n°. 74), *S. simulans* (n°. 94), *S. lugdunensis* (n°. 115), *S. haemolyticus* (n°. 137 and n°. 193), *S. hominis* (n°. 124), and *S. auricularis* (n°. 136). Except for the *S. saprophyticus* strain, all were resistant to methicillin. Within the *Enterococcus* genus, three strains belonged to the species *Enterococcus faecalis* (n°. 1a, 365, and 431), all resistant to vancomycin; strain 1a also showed resistance to teicoplanin. Three other strains were *Enterococcus faecium* (n°. 185, 186, and 300), all resistant to teicoplanin and vancomycin.

Among Gram-negative bacteria, three strains of *Escherichia coli* were studied, including a *K. pneumoniae* carbapenemase of class A-producing strain and a strain producing metallo-β-lactamase of the NDM type (New Delhi). The remaining three strains were *Pseudomonas aeruginosa*, one of which was a carbapenemase producer isolated from a patient with cystic fibrosis, and one other was resistant to carbapenem and colistin.

All the strains were obtained from the School of Medical and Pharmaceutical Sciences (University of Genoa) and identified by VITEK^®^ 2 (Biomerieux, Firenze, Italy) or matrix-assisted laser desorption/ionization time-of-flight (MALDI-TOF) mass spectrometric technique (Biomerieux, Firenze, Italy).

### 4.5. MW NADES Extraction

Fresh peony flowers were added to the selected eutectic system previously prepared (BG, BGS) in a 1:10 *w/w* ratio. After 5 min of sonication, the mixture was processed for 5 min under microwave activation at a temperature setpoint of 75 °C. After a simple filtration, the obtained sample solution was collected and kept at 4 °C until use [36].

### 4.6. Total Phenolic Content (TPC)

The total phenolic content of each sample was assessed by the Folin–Ciocalteu UV/VIS spectrophotometric method, using gallic acid as the reference standard [28]. TPCs were calculated from a calibration curve obtained from gallic acid standard solutions in concentrations ranging from 20 to 80 mg/L (R_2_ = 0.9988).

Each reaction mixture, analyzed in cuvettes, consisted of 400 µL of MilliQ water, 80 µL of sample or standard solution, 40 µL of Folin–Ciocalteu reagent, and 480 µL of a 10.75% sodium carbonate (Na_2_CO_3_) solution. After incubating the mixtures for 30 min at room temperature, the absorbance was measured at 760 nm [46].

Values are expressed as mg equivalents of gallic acid per gram of fresh peony petals (mg GAE/g). The results were derived from triplicate analyses of each extract (*n* = 3), normalized against a negative control of the corresponding eutectic solvent, and values are given ± standard deviation (SD).

### 4.7. Radical Scavenging Activity (RSA%)

The radical scavenging activity of each extract was measured by DPPH assay, based on the bleaching rate of the stable radical 2,2-Diphenyl-1-picrylhydrazyl (DPPH), using Trolox as the reference standard [29] and obtaining a linear calibration curve ranging from 20 to 200 mg/L (R_2_ = 0.9952).

A total of 0.1 mL of sample was mixed with 3.9 mL of DPPH methanolic solution (65 μM). After mixture storage for 30 min in the dark, absorbance was measured at 516 nm. The results were calculated as Trolox equivalents in solution (mg/L), and the percentage of radical scavenging activity (RSA%) was calculated from the ratio of decreasing absorbance of sample solution (A_0_-A_S_) to absorbance of blank DPPH solutions (A_0_), as expressed in Equation (1) [47]. Each analysis was performed in triplicate (*n* = 3), and values are given ± standard deviation (SD)

RSA% = (A_0_ − As)/A_0_ × 100 (1)

### 4.8. Minimal Inhibitory Concentrations (MICs)

For all samples (PF-BG and PF-BGS) and NADESs, Minimum Inhibitory Concentrations (MICs) were assessed on 38 bacterial strains using the microdilution method, according to the guidelines of the European Committee on Antimicrobial Susceptibility Testing [31]. After overnight incubation, bacterial cultures were diluted to yield a standardized inoculum of 1.5 × 10^8^ CFU/mL. Appropriate aliquots of each suspension were added to 96-well microplates containing the same volumes of serial 2-fold dilutions ranging, respectively, from 231.5 mg/mL to 0.455 mg/mL for PF-BG and from 230.5 mg/mL to 0.455 mg/mL for PF-BGS to achieve a final bacterial concentration of about 5 × 10^5^ cells/mL. The pure NADESs employed (BG and BGS) were also assessed to evaluate the possible antibacterial activity of the solvents themselves.

After 24 h incubation at 37 °C, the MIC values were determined as the lowest concentration that inhibited visible bacterial growth in the wells, compared with the compound-free control, expressed in mg/mL. All tests were performed as three independent experiments, each carried out in triplicate, and MIC values were expressed as median/modal values.

### 4.9. Time-Kill Curves

Time-kill assays were performed on three selected MRSA strains (18, B, and ATCC 29213) for the PF-BG sample. Briefly, for each strain, a mid-log-phase bacterial culture was diluted in 10 mL of Mueller-Hinton (MH) broth containing 4×MIC of the sample to yield a final inoculum of approximately 5.0 × 10 CFU/mL; the same bacterial inoculum was added to the MH broth as a bacterial growth control. All samples were incubated at 37 °C with constant shaking for 24 h. 0.20 mL aliquots were taken from each sample after 0, 2, 4, 6, and 24 h of incubation. After appropriate dilution with a 0.9% sodium chloride solution to prevent carryover of the test compounds, the samples were seeded in MH plates and incubated for 24 h at 37 °C. Growth controls were run in parallel. The percentage of surviving bacterial cells was determined for each sampling time by comparing the number of colonies with that of the standard growth control dilutions. The results were expressed as the log10 of the number of viable bacterial cells (CFU/mL) surviving over a 24 h period. All experiments to define the time-kill curves for each strain were performed in triplicate.

### 4.10. Biofilm Inhibition/Degradation

The capability of PF-BG NADES-based extracts, BG solvents, and SDA in the inhibition of biofilm formation was evaluated on seven selected *S. aureus* MRSA strains (B, 10, 11, 18, 189, ATCC 29213, and N2) using the crystal violet (CV) assay, as described by Cramton et al. [32]. Briefly, after overnight incubation in fresh Tryptic Soy Broth medium supplemented with 0.25% glucose (TSBg), the bacterial cell suspensions were brought to a turbidity corresponding to 0.5 McFarland and subsequently diluted to a final concentration of approximately 1 × 10^5^ CFU/mL in TSBg. 200 µL of the diluted solution was transferred to each flat-bottom well of a 96-well polystyrene plate, to which different concentrations of PF-BG were added, corresponding to ¼ MIC, ½ MIC, and MIC. Untreated bacterial cultures served as controls.

After incubation at 37 °C for 24 h, the culture supernatant was gently removed, and the wells were washed three times with phosphate-buffered saline (PBS). 200 µL of 0.1% CV solution was added to the dry plate. After 20 min, the wells were washed three times with PBS, revealing the stained biofilm, which was solubilized using 200 µL of ethanol. The UV absorbance was measured with a microtiter plate reader at 570 nm, and the blank value, corresponding to the absorbance of TSBg-treated wells with CV and ethanol, was subtracted from all readings.

The percentage residual (RT) of biofilm growth was calculated using the following formula:

(At/Ac) × 100(2)

where Ac is the absorbance measured for the control wells, and At is the absorbance measured in the presence of PF-BG extract.

The percentage of inhibition growth is thus obtained as follows:

100 − RT(3)

### 4.11. GC-MS Analysis

NADES samples (1 g) were diluted with 1.2 mL of ultrapure water, then extracted with n-hexane (3 × 1 mL). The organic phase was dried over anhydrous Na_2_SO_4_ and completely evaporated using a gentle N2 stream at room temperature. The residue was dissolved in 50 μL of n-hexane before analysis. The analyses were carried out using a GC Model 6890N, coupled to a benchtop MS Agilent 5973 Network (Agilent, Santa Clara, CA, USA). Chromatographic separation was performed using an Elite-5MS (5% phenyl methyl polysiloxane) capillary column of (30 m × 0.32 mm i.d.) and film 0.32 μm thick (Agilent, Santa Clara, CA, USA). One μL aliquot of each sample was manually injected in splitless mode [35]. The oven temperature program included an initial isotherm at 40 °C for 5 min, followed by a temperature ramp to 260 °C at 40 °C/min, and a final isotherm at 260 °C for 10 min. Injector and detector temperatures were set at 250 °C and 280 °C, respectively. Mass spectra were acquired over a 40–400 amu range at 1 scan/sec with an ionizing electron energy of 70 eV. The identification of the volatile compounds was performed using Retention Indices (RI) and mass spectra according to Adams [38] by comparison with online published data [39] and with a NIST database mass spectral library [41]. The relative amount of each component was expressed as a percentage of the total peak area from GC/MS analyses of the whole extract.

### 4.12. Statistical Analysis

Microbiological analyses were assessed in triplicate, and data were subjected to analysis of variance (ANOVA) using GraphPad Prism version 8.0.0 for Windows, GraphPad Software, San Diego, CA, USA. Wherever F values were significant, the Tukey test was used for means comparison. Significance was defined at *p* < 0.001.

## 5. Conclusions

The extractive process applied can be considered sustainable, adhering to several principles of green extraction, considering the use of alternative natural solvents (NADESs—natural deep eutectic solvents), an alternative energetic source (microwave activation), and a renewable and recycled natural source (*Lactiflora pall* flowers). The HBA and HBD selected for the extractive procedure (betaine, glycerol, and sorbitol) give rise to eutectic systems characterized by good stability, safety, and intrinsic dermocosmetic properties, in addition to their high extraction efficiency. The final extract complexes obtained, consisting of the active metabolites of the plant and NADES itself, were then easily evaluated in their entirety, disclosing interesting antioxidant activity, antimicrobial properties, and scented character.

The outcomes demonstrate good stability over time, thus positioning the complex as a promising candidate as a natural, multifunctional, and ready-to-use ingredient for cosmetic application, contingent upon subsequent comprehensive assessments of stability and safety. The NADES-based extracts, with concurrent antioxidant activity, antimicrobial functionalities, and olfactive properties, could be directly enclosed in the final products, avoiding further manipulation or purification steps and increasing the naturalness and performance of the formulations.

## Figures and Tables

**Table 1:** Total phenolic content (TPC) and radical scavenging activity (RSA%) of NADES samples (PF-BG, PF-BGS) by means of DPPH (2,2-Diphenyl-1-picrylhydrazyl) and Folin–Ciocalteu assays. Results are expressed as mean values ± standard deviation (SD) of three independent experiments (*n* = 3).

NADES Sample	TPC (mg GAE/g ± SD)	RSA % (±SD) *
PF-BG	34.38 ± 0.03	87.50 ± 0.06
PF-BGS	33.78 ± 0.02	86.98 ± 0.06

GAEs = gallic acid equivalents; * positive control = Trolox.

**Table 2:** Minimal Inhibitory Concentrations (MICs) expressed as mg/mL of NADESs (BG, BGS) and NADES-based extracts (PF-BG and PF-BGS) against Gram-positive bacterial species. Experiments were carried out in triplicate. The degree of concordance in all the experiments was 3/3. Variation among triplicate samples was less than 10%.

Bacterial Species	Strain	MIC (mg/mL)			
		BG	PF-BG	BGS	PF-BGS
*S. aureus*	10 *	-	7.2	-	28.8
	11 *	-	7.2	-	28.8
	18 *	-	7.2	-	57.6
	187 *	-	7.2	-	14.4
	189 *	-	7.2	-	28.8
	A *	-	14.5	-	28.8
	B *	-	14.5	-	28.8
	C *	-	14.5	-	14.4
	D *	-	14.5	-	14.4
	N2	-	7.2	-	28.8
	ATTC 29213	-	7.2	-	14.4
*S. epidermidis*	22 *	-	7.2	-	28.8
	180 * ^§^	-	3.6	-	28.8
	181 * ^§^	-	3.6	-	14.4
	222 * ^§^	-	3.6	-	28.8
	198 * ^§^	-	7.2	-	14.4
	216 * ^§^	-	7.2	-	14.4
*Coagulase-negative* *Staphylococci*	*S. saprophyticus* 41	-	0.9	-	7.2
	*S. capitis* 71 *	-	1.8	-	7.2
	*S. warneri* 74 *	-	0.9	-	7.2
	*S. simulans* 94 *	-	7.2	-	28.8
	*S. lugdunensis* 115 *	-	14.4	-	57.6
	*S. haemolyticus* 137 *	-	14.4	-	57.6
	*S. haemolyticus* 193 *	-	14.4	-	28.8
	*S. hominis* 124 *	-	0.9	-	7.2
	*S. auricularis* 136 *	-	0.9	-	3.6
*E. faecalis*	1a ^vt^	-	28.8	-	-
	365 ^v^	-	28.8	-	-
	431 ^v^	-	28.9	-	-
*E. faecium*	185 ^vt^	-	57.8		-
	186 ^vt^	-	57.8	-	-
	300 ^vt^	-	57.8	-	-

* = methicillin resistance; § = linezolid resistance; v = vancomycin resistance; t = teicoplanin resistance.

**Table 3:** Minimal Inhibitory Concentrations of SDA solutions (in BG), expressed as mg/mL, against selected *S. aureus* strains, in comparison to BG solvent. Experiments were carried out in triplicate. The degree of concordance in all the experiments was 3/3. Variation among triplicate samples was less than 10%.

Species	Strain	MIC (mg/mL)	
		BG	SDA
***S. aureus***	10 *	-	3.8
	11 *	-	7.6
	18 *	-	3.8
	189 *	-	7.6
	B *	-	7.6
	N2	-	7.6
	ATTC 29213	-	7.6

* = methicillin resistance.

**Table 4:** Inhibition percentage of biofilm formation (±SD) of PF-BG at different concentrations (¼ MIC, ½ MIC, and MIC).

*S. aureus* Strain	% Inhibition of Biofilm Formation		
	¼ MIC	½ MIC	MIC
B *	90.4 ± 2.1	90.1 ± 1.8	93.1 ± 1.8
18 *	80.7 ± 4.2	66.6 ± 9.1	65.5 ± 5.2
10 *	64.3 ± 13.1	57.7 ± 18.0	51.6 ± 17.3
11 *	74.6 ± 6.4	54.1 ± 24.8	28.6 ± 27.4
189 *	81.9 ± 4.6	71.5 ± 9.0	63.4 ± 12.0
**ATCC 29213**	77.3 ± 10.4	54.9 ± 10.4	32.3 ± 21.6
**N2**	84.6 ± 3.9	77.5 ± 11.1	67.4 ± 14.6

* = methicillin resistance.

**Table 5:** Inhibition percentage of biofilm formation of SDA at different concentrations (¼ MIC, ½ MIC, and MIC).

*S. aureus* Strain	% Inhibition of Biofilm Formation		
	¼ MIC	½ MIC	MIC
B *	84.5 ± 9.9	84.5 ± 10.3	92.0 ± 8.9
18 *	88.5 ± 4.7	93.7 ± 2.5	95.4 ± 1.2
10 *	78.5 ± 10.0	93.6 ± 2.9	92.9 ± 4.1
11 *	70.4 ± 12.1	68.5 ± 17.5	81.7 ± 5.9
189 *	81.9 ± 15.9	89.6 ± 7.0	90.9 ± 4.2
**ATCC 29213**	73.1 ± 12.0	85.5 ± 6.5	86.3 ± 4.3
**N2**	85.4 ± 6.9	82.5 ± 8.7	87.9 ± 4.0

* = methicillin resistance.

**Table 6:** Retention Indices, relative percentage, identification method, and olfactory description of the aromatic compounds of interest.

Compound ^a^	RI ^b^	RI ^c^	Mean % (±SD)	Identif. Method ^d^	Olfactive Description
Limonene	1014	1015	0.02 ± 0.08	NIST, RI	lemon, orange
2-phenylethanol	1139	1139	0.62 ± 0.12	NIST, RI	honey, spice, rose, lilac
Citronellol	1230	1229	0.15 ± 0.09	NIST, RI	rose

^a^ Compounds listed in order of their elution on an Elite-5 column. ^b^ RI = Retention Indices according to Adams [38] and with online published data [39]. ^c^ RI = Retention Indices determined on an Elite-5 column using a homologous series of n-hydrocarbons [40]. ^d^ Method of identification: NIST = National Institute of Standards and Technology database library [41]; RI = Retention Indices, in agreement with literature values [38,40].

**Table 7:** TPC and RSA% values of the PF-BG sample after 90 days (t_90d_) and 9 months (t_180d_). The initial value (t_0_) is also reported for comparison. Mean values (*n* = 3) ± standard deviation (SD).

PF-BG	TPC (mg GAE/g ± SD)	RSA % (±SD)
t_0_	34.38 ± 0.03	87.50 ± 0.06
t_90_	34.31 ± 0.05	87.30 ± 0.03
t_180_	32.75 ± 0.02	85.80 ± 0.04

**Table 8:** MIC values for the PF-BG sample at time zero (t_0_) and after 90 days (t_90_). Experiments were carried out in triplicate. The degree of concordance in all the experiments was 3/3. Variation among triplicate samples was less than 10%.

Strain	MIC t_0_	MIC t_90_
*S. aureus* 18 *	7.2	7.2
*S. epidermidis* 22 *	7.2	7.2
*E. faecalis* 1 °	14.4	14.4
*E. faecium* 185 °	28.8	28.8

* resistance to methicillin, ° resistance to vancomycin and teicoplanin.

## Data Availability

Data are available on request from the corresponding author.

## Abbreviations

The following abbreviations are used in this manuscript:

NADES	Natural deep eutectic solvent
BG	Betaine/Glycerol NADES
BGS	Betaine/Glycerol/Sorbitol NADES
PF-BG	Betaine/Glycerol NADES-based extract sample
PF-BGS	Betaine/Glycerol/Sorbitol NADES-based extract sample
MW	Microwaves
MICs	Minimal Inhibitory Concentrations
NIST	National Institute of Standards and Technology
RI	Retention Indices
STD	GC-MS Standard
GAEs	Gallic acid equivalents
